# Acapsular *Staphylococcus aureus* with a non-functional *agr* regains capsule expression after passage through the bloodstream in a bacteremia mouse model

**DOI:** 10.1038/s41598-020-70671-1

**Published:** 2020-08-24

**Authors:** Carlos M. Suligoy, Rocío E. Díaz, Ana-Katharina Gehrke, Natalie Ring, Gonzalo Yebra, Joana Alves, Marisa I. Gómez, Sindy Wendler, J. Ross FITZGERALD, Lorena Tuchscherr, Bettina Löffler, Daniel O. Sordelli, Mariángeles Noto Llana, Fernanda R. Buzzola

**Affiliations:** 1 Instituto de Investigaciones en Microbiología y Parasitología Médica (IMPaM UBA-CONICET), Buenos Aires, Argentina; 2 Departamento de Investigaciones Biomédicas y Biotecnológicas, Centro de Estudios Biomédicos, Biotecnológicos, Ambientales y de Diagnóstico (CEBBAD), Universidad Maimónides and CONICET, Buenos Aires, Argentina; 3 The Roslin Institute, Royal (Dick) School of Veterinary Medicine, University of Edinburgh, Easter Bush Campus, Edinburgh, UK; 4 Institute of Medical Microbiology, Jena University Hospital, Jena, Germany

**Keywords:** Microbiology, Pathogenesis

## Abstract

Selection pressures exerted on *Staphylococcus aureus* by host factors during infection may lead to the emergence of regulatory phenotypes better adapted to the infection site. Traits convenient for persistence may be fixed by mutation thus turning these mutants into microevolution endpoints. The feasibility that stable, non-encapsulated *S. aureus* mutants can regain expression of key virulence factors for survival in the bloodstream was investigated. *S. aureus agr* mutant HU-14 (IS*256* insertion in *agrC*) from a patient with chronic osteomyelitis was passed through the bloodstream using a bacteriemia mouse model and derivative P3.1 was obtained. Although IS*256* remained inserted in *agrC*, P3.1 regained production of capsular polysaccharide type 5 (CP5) and staphyloxanthin*.* Furthermore, P3.1 expressed higher levels of *asp23/*SigB when compared with parental strain HU-14. Strain P3.1 displayed decreased osteoclastogenesis capacity, thus indicating decreased adaptability to bone compared with strain HU-14 and exhibited a trend to be more virulent than parental strain HU-14. Strain P3.1 exhibited the loss of one IS*256* copy, which was originally located in the HU-14 noncoding region between *dnaG* (DNA primase) and *rpoD* (*sigA)*. This loss may be associated with the observed phenotype change but the mechanism remains unknown. In conclusion, *S. aureus* organisms that escape the infected bone may recover the expression of key virulence factors through a rapid microevolution pathway involving SigB regulation of key virulence factors.

## Introduction

*Staphylococcus aureus* is a transient, sometimes permanent member of the human microbiota in the nares and skin of a significant number of healthy individuals. As predisposing conditions emerge in the host, this opportunistic species may cause infections with different severity, ranging from mild skin and soft tissue infection to severe disseminated disease^1,2^. Treatment of *S. aureus* infections is hampered by widespread dissemination of methicillin-resistant *S. aureus* (MRSA)^3^ and by the frequent emergence of *S. aureus* with low level resistance to vancomycin^4^. Whereas MRSA was primarily considered a nosocomial pathogen^5^, it is now unanimously accepted that MRSA also affects individuals of the general community with no previous exposure to health care settings^6,7^. Up to 1.5–2% of patients receiving an orthopedic prosthetic device becomes infected and a significant number of these infections are caused by *S. aureus*. Most osteomyelitis caused by *S. aureus* become refractory to antibiotic treatment soon after bacteria settles on the prosthetic device surface and in bone tissue^1^. *S. aureus* has the ability to swiftly adapt to the conditions encountered at the infection niche by adjusting its metabolism and/or regulating the expression of virulence factors required for successful establishment at the incipiently colonized tissue. Once *S. aureus* has adapted to the microenvironment certain traits may be fixed by mutations, which occur as osteomyelitis becomes chronic^8,9^. Indeed, selection pressure exerted by a vast number of yet undefined host factors would permit the emergence of bacterial variants more suitable to evade immune defense mechanisms and cause infection refractory to antibiotic treatment in the absence of genes that code antibiotic resistance.

*Staphylococcus aureus* possesses a vast repertoire of virulence and immune evasion factors that facilitates its dual life-style as either a commensal or a pathogen^1,10^. More important, *S. aureus* displays a complex regulatory network, composed of a number (as yet not fully known) of genes which allow the crosstalk between regulators^11^ thus permitting this species to rapidly switch on and off virulence factors to adapt to and survive in changing microenvironments. One of the strategies to investigate which factors may be up- or down-regulated during adaptation is to assess whether any of these factors is fixed by mutation during chronic infection. Among these factors, loss of short sequence–repeats in the protein A Xr region, small colony variant (SCV) emergence and loss of capsular polysaccharide (CP) expression can be mentioned^12–15^. Furthermore, we were able to demonstrate that the loss of CP expression due to a mutation in the *agr* occurs during chronic osteomyelitis^9^. This finding suggested that loss of RNAIII expression may yet be another endpoint in microevolution since *agr* regulates the expression of a vast number of virulence factors.

CP5 and CP8 are produced by a 75 to 80% of *S. aureus* isolates from humans and play a significant role in the pathogenesis of staphylococcal infections^16^. Isolates of *S. aureus* that fail to produce CP5 or CP8 and that produce non-mucoid colonies on solid media are defined as non-typeable (NT) regardless the mechanism responsible for the lack of CP expression^17^. CP5 and CP8 are virulence factors that permit *S. aureus* to avoid phagocytosis and facilitate bloodstream dissemination^18,19^. Once established in the bone of the patient with chronic osteomyelitis it appears advantageous for *S. aureus* to loose CP expression^15^ to remain undisturbed within the infected bone. To support the hypothesis that loss of CP expression may initially be due to regulation, in a very recent study it was demonstrated that *S. aureus* can express CP in vivo, even though production in vitro cannot be demonstrated^20^. These findings showed that *S. aureus* can switch on or off CP expression according to the in vivo microenvironment surrounding bacteria. In the present study, we investigated whether *S. aureus* can regain the ability to produce CP, even in the presence of a mutated non-functional Agr, when it is transferred from infected bone to the bloodstream.

## Results

### Mouse passages of strain HU-14

**Figure 1:**
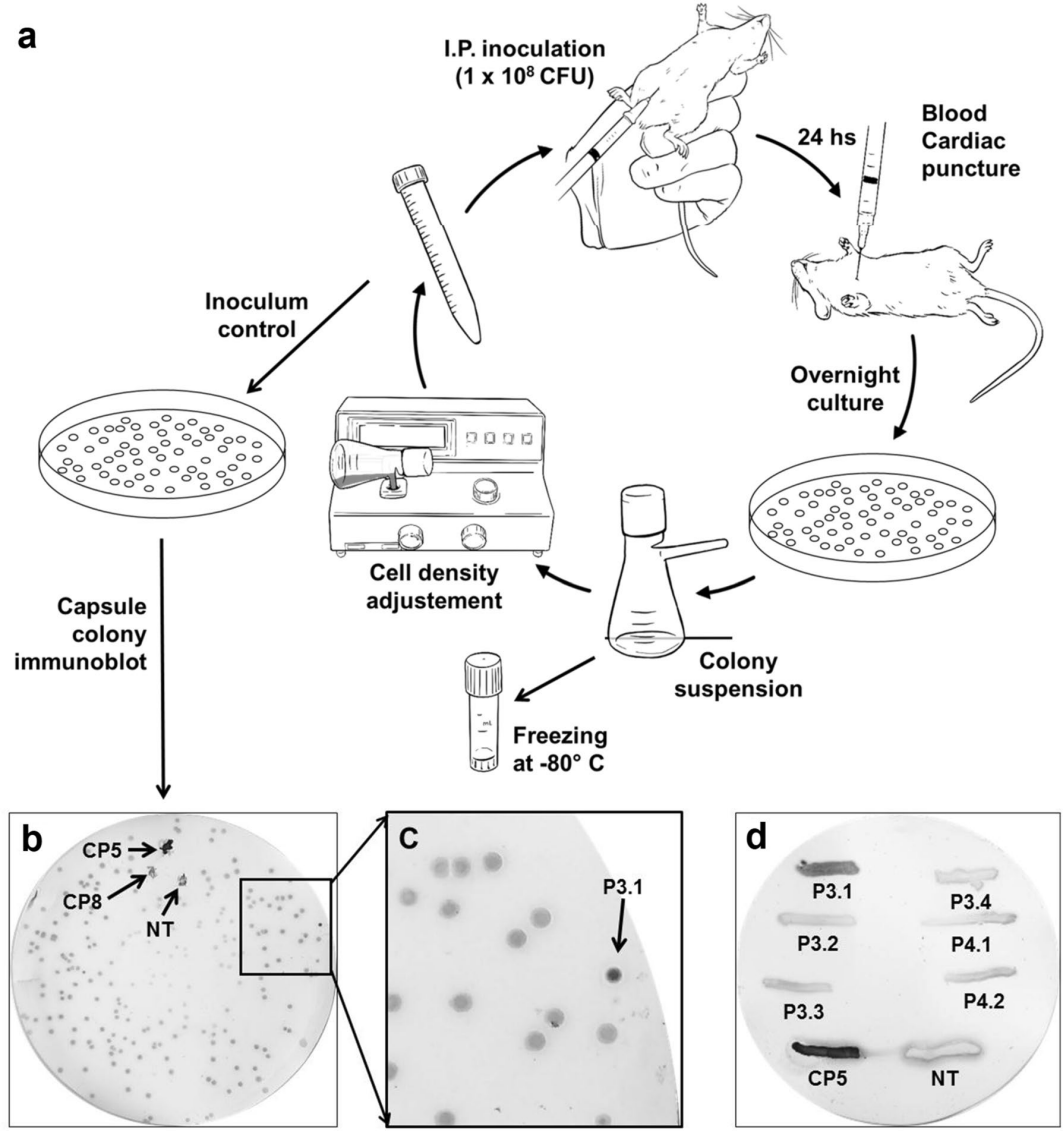
(**a**) Mouse experiment flowchart. Mice were injected by the i.p. route with 1 × 10^8^ CFU of *S. aureus* HU-14. After 24 h mice were sacrificed and blood obtained by cardiac puncture. Blood was plated quantitatively and after an overnight culture all colonies from a plate were harvested and suspended in PBS, and the suspension adjusted to a density of 1 × 10^9^ per ml for further injection to another group of mice. Suspension aliquots were plated for CFU count and colony immunoblots were performed on the grown TSB plates to detect the emergence of any colony producing CP5. (**b**) Colony immunoblot of strain HU-14 after three passages through mice. The arrows indicate strains Reynolds CP5, Reynolds CP8 and Reynolds NT. These three control strains were added by impregnation of the blot membrane. (**c**) Magnification of the photograph shown in (**b**). The arrow indicate colony #1, later identified as P3.1. (**d**) P3.1 was confirmed as CP5. Positive control was strain Reynolds CP5 (lower left strike). Negative control was Reynolds NT (lower right strike).

Strain HU-14 lacks CP5 expression due to an IS*256* insertion in *agrC*. Strain HU-14 was passed through blood using the bacteremia mouse model and the emergence of colonies with positive reaction to anti-CP5 immune serum was monitored by colony immunoblot assay. After passage #3 (Fig. 1a) a colony (#1) reacting to anti-CP5 was pinpointed (Figs. 1b and 1c), which was recorded as strain P3.1. Other suspicious colonies from the same passage were tested for CP expression but only P3.1 was confirmed as CP5 positive (Fig. 1d). The P3.1 colony was amplified and subcultured several times to assess phenotype stability and after 7 passages on TSA the CP5 phenotype remained stable.

### Isolate P3.1 characterization

**Figure 2:**
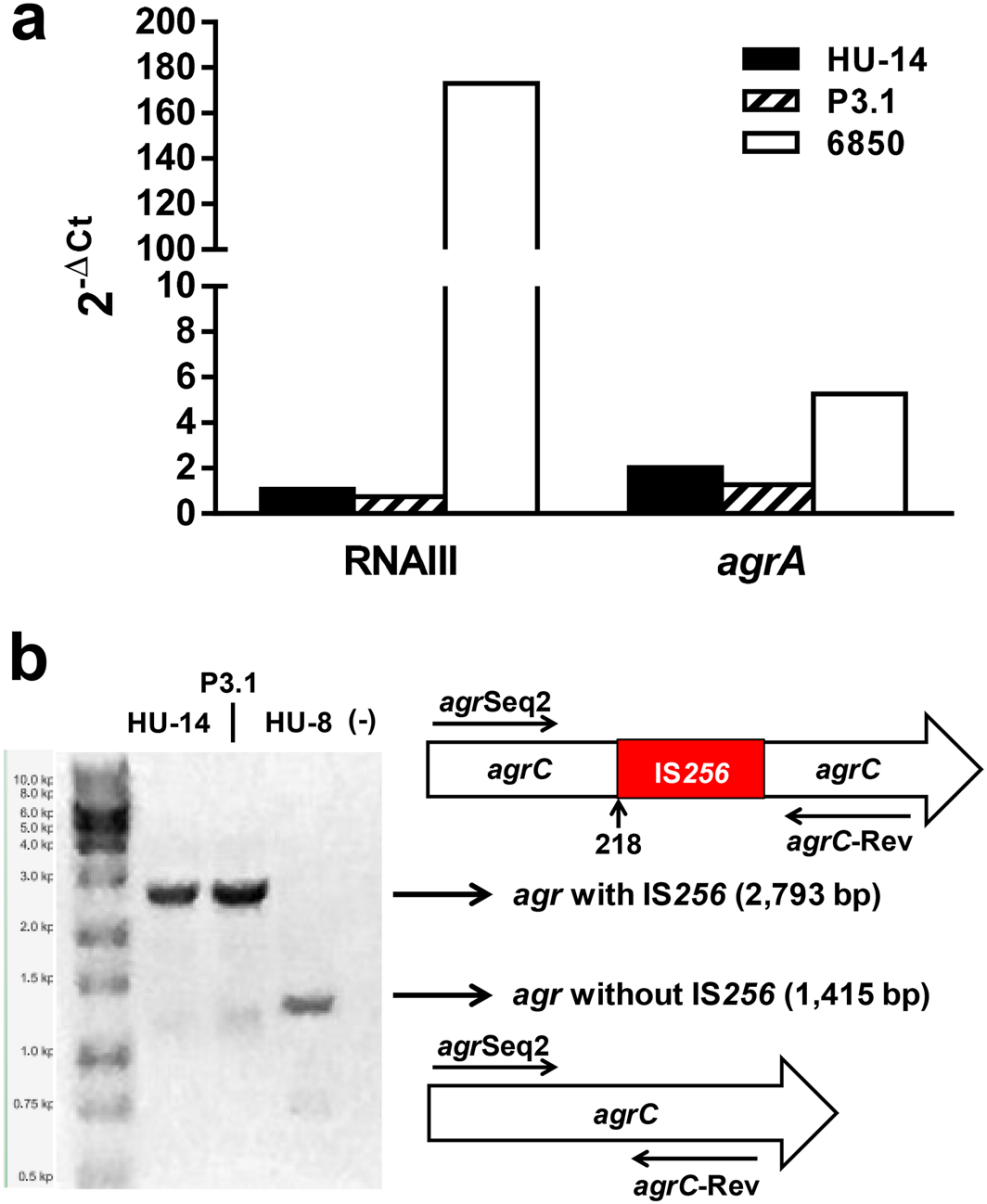
(**a**) Quantitative real time PCR of RNAIII and *agrA* transcripts from strains HU-14 and derivative P3.1. *S. aureus* strain 6,850 was used as reference. Changes in gene expression are shown as normalized mean fold change 2^-ΔCt^. Data were normalized to *16S* expression. The data represent the mean of duplicate measurements from 3 independent experiments. (**b**) Electrophoretic run of *agrC* PCR amplicons. HU-14 and P3.1 rendered amplicons of identical size, larger than that obtained from control strain HU-8 with a conserved *agrC*. Lane (-) contains no DNA. The original gel image is shown in Suppl. Figure 3. The result obtained shows that the IS*256* insertion remains in place within *agrC* in derivative P.3.1.

**Figure 3:**
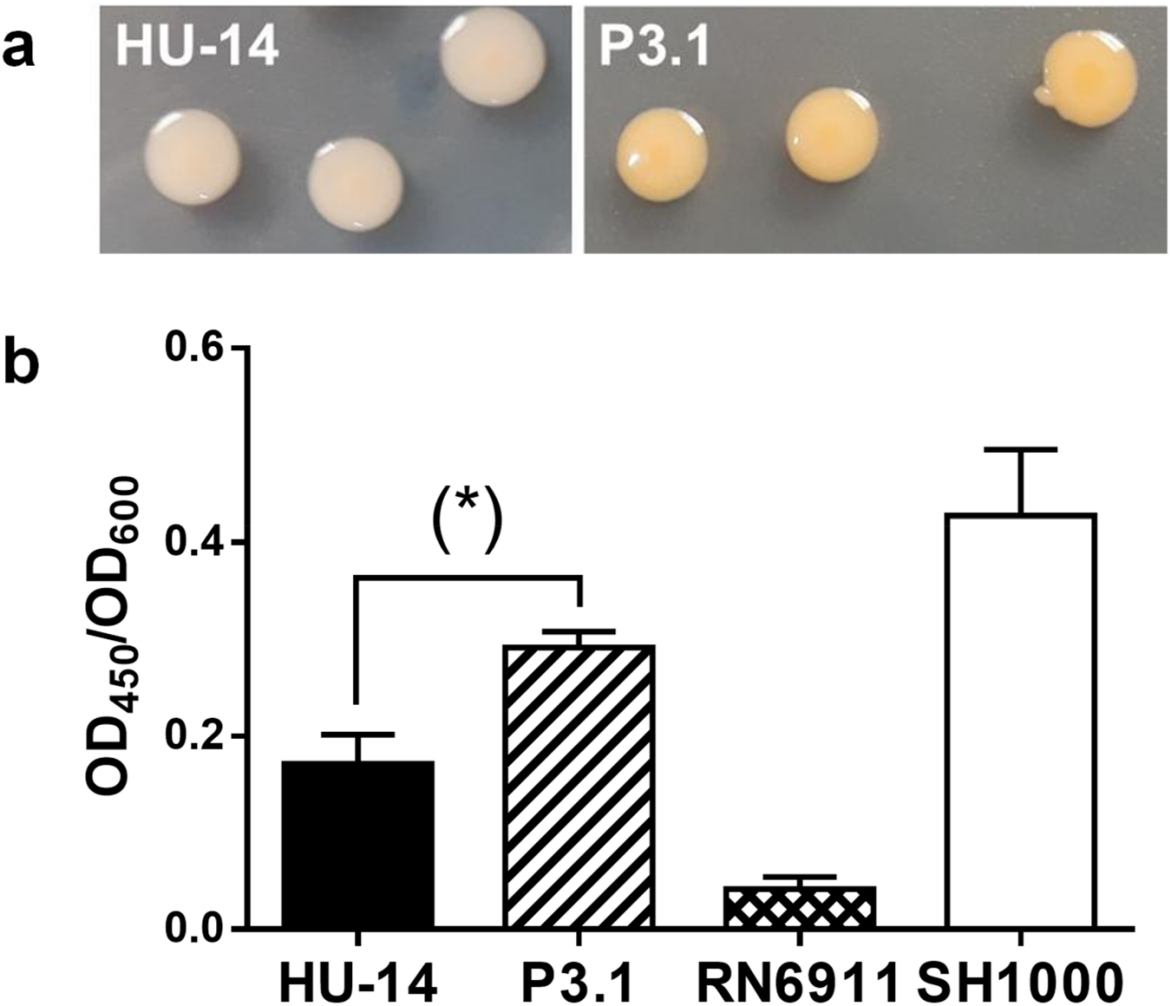
(**a**) Strain HU-14 and P3.1 colonies on TSA showing the different pigmentation level. Panel b: pigments were extracted *S. aureus* strains with methanol and the optical density ratios at 450 nm/600 nm were measured. Sample size in each column varied from 4 to 18. The P3.1 methanolic extract produced a significantly higher absorbance when compared with the parental HU-14 strain. (*) p = 0.0121, Student *t* test for unpaired samples. *S. aureus* RN6911 does not produce staphyloxanthin and SH1000 is a heavy staphyloxanthin producer strain.

Phenotypic characterization revealed that strains HU-14 and P3.1 have almost identical mean generation times (MGT) (40 min and 39 min, respectively). Biofilm production was significantly higher (p < 0.0001, Student *t* test) in the P3.1 derivative [OD_**B**_/OD_**G**_ = 0.60 ± 0.02 (n = 42)] when compared with the parental HU-14 strain [OD_**B**_/OD_**G**_ = 0.35 ± 0.01 (n = 42)], as assessed by the crystal violet test. Total hemolytic activity was negative for both strains as well as for alpha- and beta-hemolysins. Proteolytic activity was negative for both strains too. Both strains were vancomycin susceptible. Transcriptional evaluation of the Agr system in HU-14 and P3.1 revealed that the expression of RNAIII and *agrA* was still abolished in P3.1 as ascertained through qRT-PCR (Fig. 2a). Amplification of the P3.1 *agrC* gene produced an amplicon of identical size (2,793 bp) to that observed in HU-14, which suggested that IS*256* remained positioned in the *agrC* gene of isolate P3.1 (Fig. 2b). Sequence analysis revealed that the insertion site of IS*256* in *agrC* was identical in both strains and positioned after base 218. In synthesis, *S. aureus* regained CP expression in the absence of a functional Agr system (lack of RNAIII and *agrA* expression). Another noticeable phenotypic feature was that P3.1 produced the classical yellowish-golden colonies that give the name to this bacterial species whereas its parental HU-14 strain produced whitish colonies on TSA (Fig. 3a). Methanolic extracts from HU-14 exhibited a significantly lower absorbance at 450 nm when compared with those of P3.1, which suggested that P3.1 produced increased levels of staphyloxanthin (Fig. 3b).

### Whole genome sequence analysis

**Figure 4:**
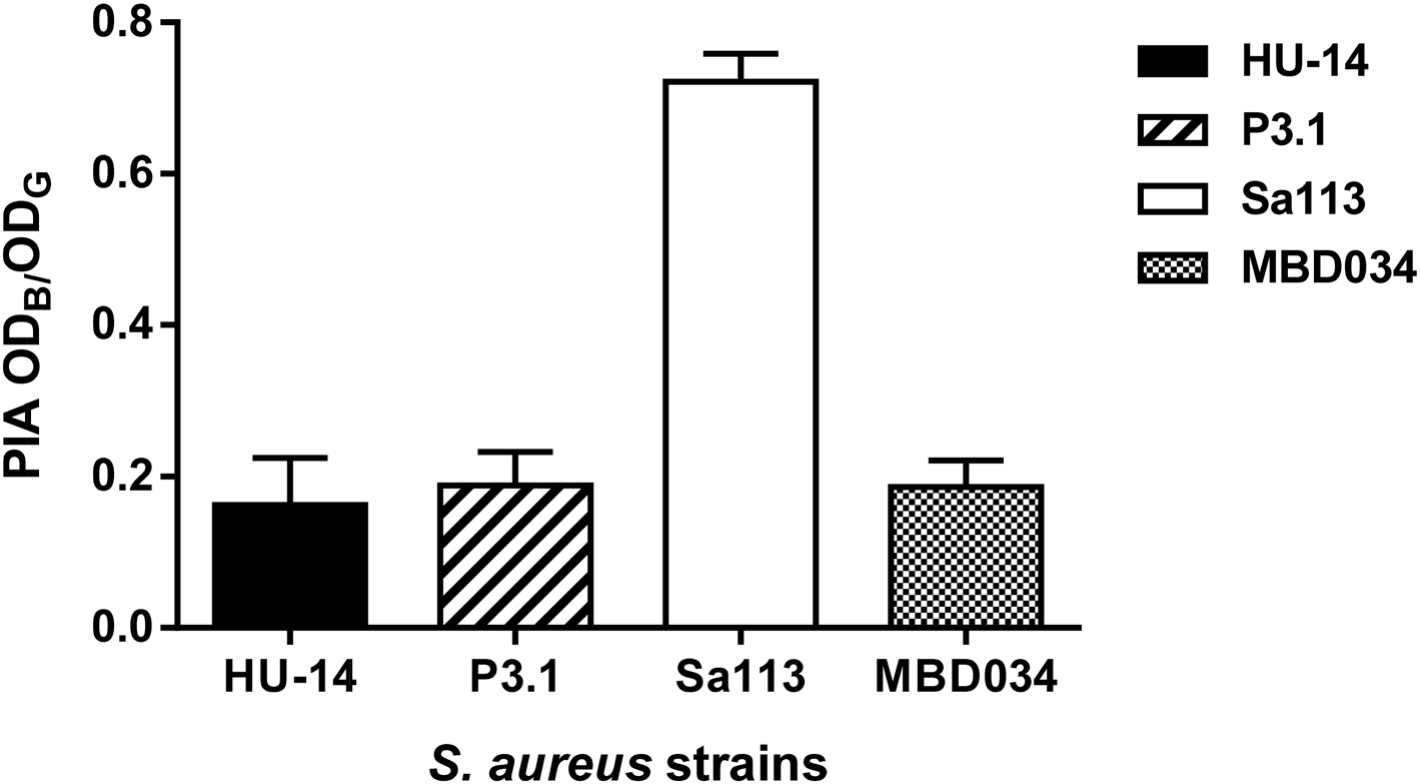
PIA in biofilms produced by *S. aureus* HU-14 and P3.1. Strains SA113 and MBD034 were included as positive standard and negative control, respectively. Each bar represents the arithmetic mean ± SEM from 4 to 6 wells from 3 separate experiments. PIA production values are the OD at 595 nm of crystal violet (OD_**B**_) relative to the final culture density (OD_**G**_) after 24 h incubation. There was no significant difference between HU-14 and P3.1 (ordinary one-way ANOVA, Tukey´s test, Brown-Forsythe post-test).

Analysis using Illumina short read sequences confirmed that HU-14 and P3.1 were isogenic, with only one single nucleotide polymorphism (SNP) in P3.1. It consisted of replacement of C by T in position 589 of the *icaA* gene, leading to a change from alanine to threonine in position 197. Phenotypical analysis of the *icaADBC* product, namely the polysaccharide intercellular adhesin (PIA) revealed negligible PIA production with no significant differences between HU-14 and P3.1 (Fig. 4). In addition to *agrC*, which was mutated by the IS*256* insertion in both strains, further short read sequence analysis determined that no genomic modification of any other known major regulator occurred which may explain the P3.1 capsulated phenotype. Furthermore, no SNP was found in the intergenic regions which may affect the expression of any regulator.

Pacific Biosciences sequencing was performed to assess whether there was any structural difference between the HU-14 and P3.1 genomes. Hybrid Unicycler assembly of PacBio and Illumina reads resulted in two genome fragments. The smaller fragment in the HU-14 and P3.1 hybrid Unicycler assemblies was a circular sequence that shared 99.98% and 99.80% identity respectively with plasmid pCM05 (PLSDB database). Annotation with Prokka using the pCM05 plasmid from the NCBI GenBank Database (NC_013332.1) as a reference and SnapGene side-by-side analysis with the pCM05 GenBank file confirmed that the circular sequence was indeed pCM05 in both assemblies, and contained all the same genes and restriction sites. The plasmid sequences were also aligned using ProgressiveMaude, but these did not show any rearrangements.

The size of the larger fragment (2,972,382 bp and 2,971,047 bp in HU-14 and P3.1, respectively) was consistent with that of the *S. aureus* chromosome. Whole genome alignment of the chromosome sequences was performed using progressiveMauve, showing no chromosomal rearrangements between the two. However, annotation with Prokka revealed that P3.1 has lost one IS*256* copy originally located in the non-coding region downstream of DNA primase (*dnaG*, LJDFIFNA_00432) and upstream of RNA polymerase sigma factor RpoD (rpoD, LJDFIFNA_00434) (*sigA*) (Suppl Fig. 1). IS*256* excision in P3.1 was clean and left a conserved region between *dnaG* and *rpoD* identical to those of N315, USA300 and other ST5 isolates from our collection.

Interestingly, when both hybrid assembly and only short read analysis was performed the IS*256* was definitively missing in P3.1 but such excision was not pinpointed by performing long read analysis only. Finally, SNP-calling between the two closed hybrid genomes was carried out using Snippy, with the Prokka-annotated HU-14 closed reference genome and the P3.1 Illumina reads, and vice-versa. This revealed the same single SNP as the same analysis using just the Illumina reads: an inconsequential change from alanine in HU-14 to threonine in P3.1, as described above.

### Regulation of CP5 expression

**Figure 5:**
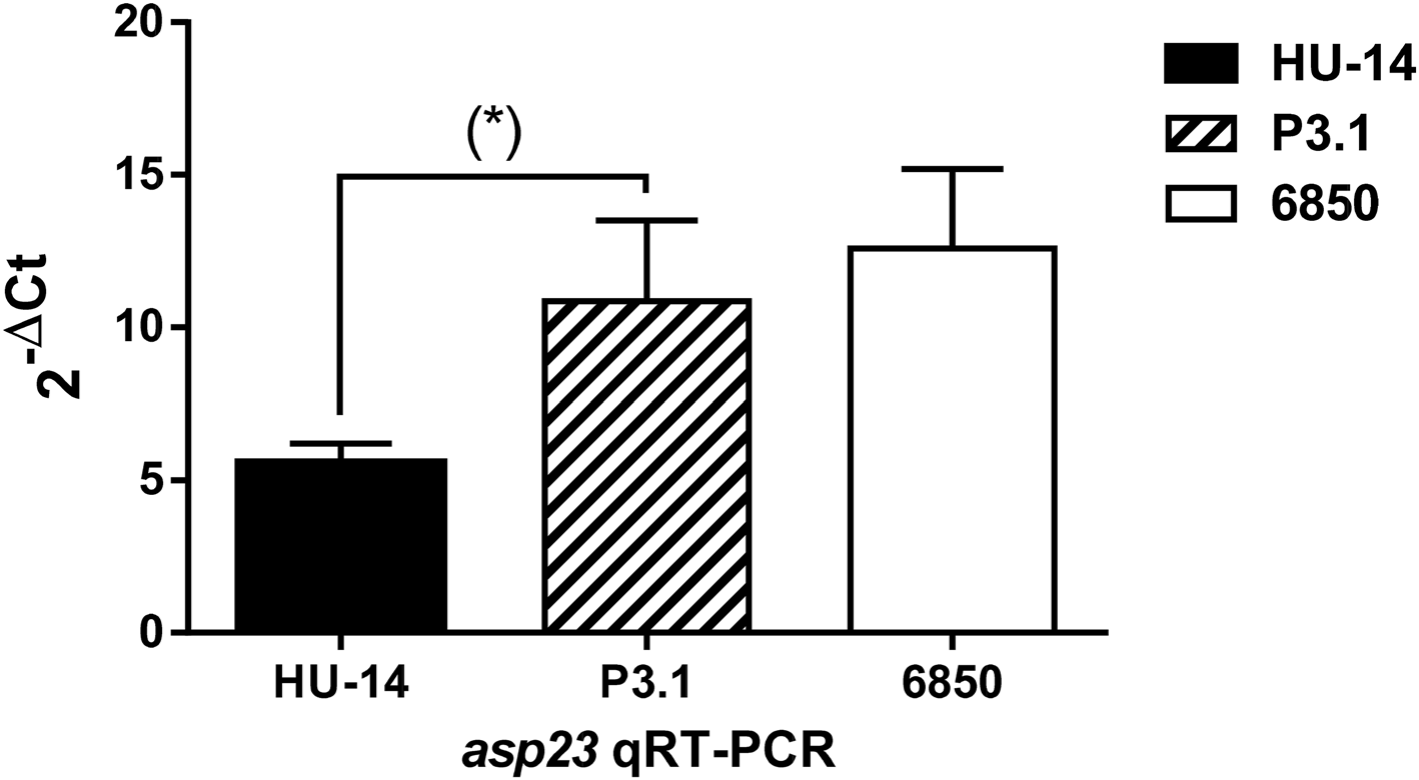
Quantitative real time PCR of *asp23* and *sigB* transcripts from strains HU-14 and derivative P3.1. *S. aureus* strain 6850 was used as reference. Changes in gene expression are shown as normalized mean fold change 2^-ΔCt^. Data were normalized to *16S* expression. The data represent the median of duplicate measurements from 3 independent experiments. (*) Significant difference with p = 0.0070 (Mann–Whitney test).

It is known that regulation of CP5(8) expression in *S. aureus* is complex and involves a number of redundant mechanisms that act in concert. Evaluation of main *S. aureus* regulators *sae* and *sarA* at the transcriptional level by qRT-PCR revealed that no functional modification occurred (data not shown). However, transcription of *asp23*, a gene that is solely regulated by SigB, was significantly increased in P3.1 when compared with HU-14 (Fig. 5). Since staphyloxanthin production by *S. aureus* is positively regulated by SigB, the increased pigmentation displayed by P3.1 supports the hypothesis that *S. aureus* P3.1 reacquired the capacity to produce CP5 through a mechanism related to recovery of SigB functionality, in the absence of an operative Agr system.

### Virulence study

The virulence of strain HU-14 and derivative P3.1 was tested in a mouse model of intraperitoneal injection. Mortality 5 days after challenge with P3.1 was higher than that in the group challenged with HU-14 but was not significantly different (Suppl Fig. 2a). Body weight was recorded over 5 days after challenge and the results showed that mice challenged with the P3.1 derivative lost more weight than those injected with the parental strain HU-14, but the differences were again not significant (Suppl Fig. 2b). Interestingly, mice challenged with P3.1 exhibited more prominent signs of distress, such as hunched posture and spiky fur, when compared with those challenged with HU-14.

### Osteoclastogenesis and cytokine production studies

**Figure 6:**
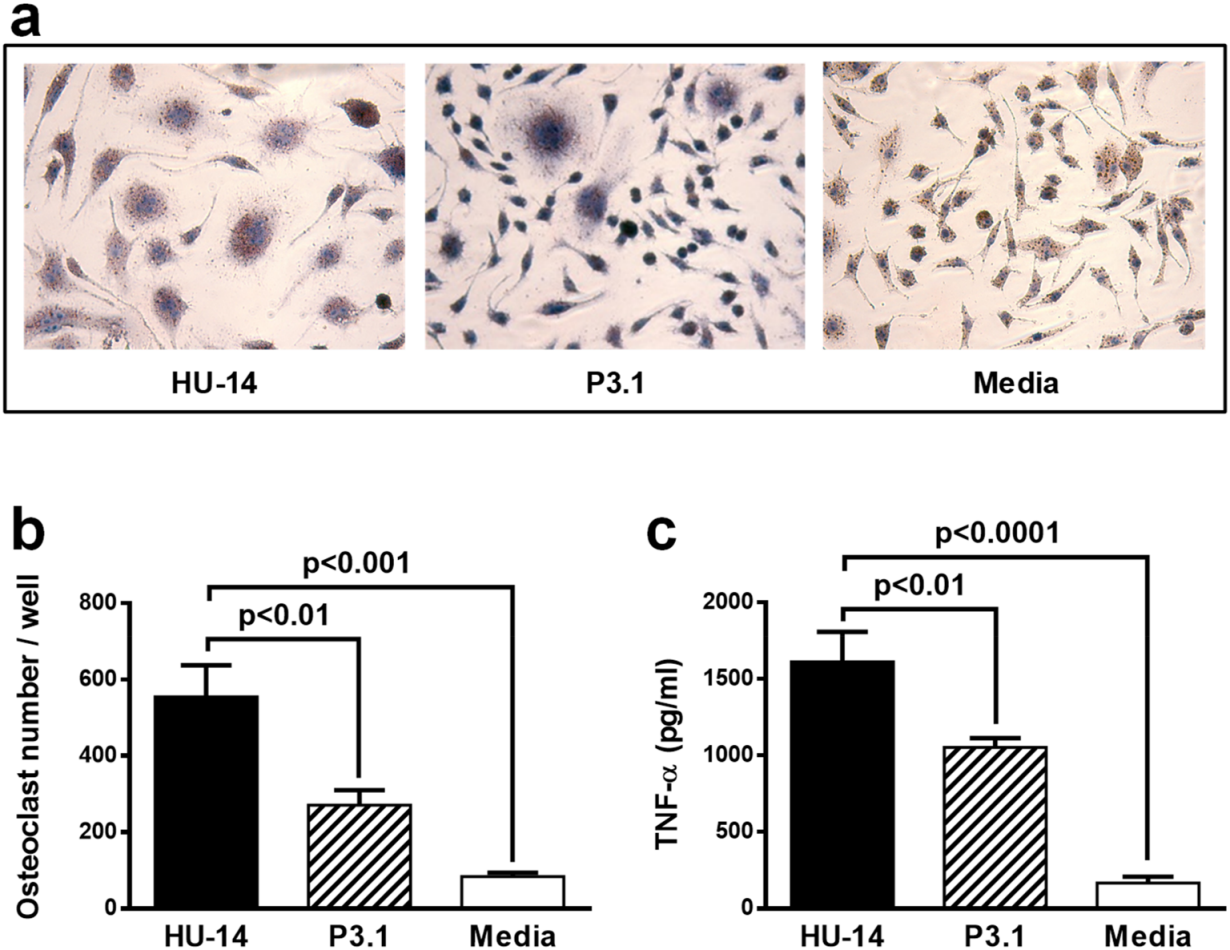
(**a**,**b**) RAW 264.7 cells were stimulated with the *S. aureus* HU-14 isolate or its derivative P3.1 (Heat-killed bacteria, 10^7^ CFU/ml) for 48 h. Media alone was used as negative control. (**a**) Photographs at 20 × magnification. (**b**) The number of mature osteoclasts was quantified by light microscopy using TRAP staining. TRAP + cells with 3 or more nuclei were considered mature osteoclasts. The mean and standard error of cumulative data from 3 independent experiments (6 wells) is shown. Levels of significance were obtained by ordinary one-way ANOVA (Tukey´s multi comparisons test, Brown-Forsythe post-test). (**c**) TNF-α production was quantified by ELISA in the culture media. The mean and standard error of cumulative data from 3 independent experiments (6 wells) is shown. Levels of significance were obtained by ordinary one-way ANOVA (Tukey’s multi comparisons test, Brown-Forsythe post-test).

The differential capacity of strains HU-14 and P3.1 to trigger osteoclastogenesis was investigated in vitro. Osteoclast precursors were stimulated with *S. aureus* in the presence of M-CSF. Forty-eight hours after stimulation cells were fixed and multinucleated TRAP ( +) cells were counted. Rapid differentiation into osteoclasts was induced by HU-14 with formation of multinucleated cells (3 or more nuclei) (Fig. 6a,b). In contrast, significantly decreased numbers of osteoclasts were observed when the osteoclast precursors were stimulated with P3.1 (Fig. 6a,b). *S. aureus* HU-14 and its P3.1 derivative were tested for their capacity to induce the production of TNF-α, a proinflammatory cytokine involved in osteoclastogenesis, by osteoclast precursors in culture. The P3.1 derivative exhibited a decreased capacity to trigger TNFα secretion (Fig. 6c).

## Discussion

*Staphylococcus aureus* has the potential to adapt to diverse niches with defined microenvironments^21^. In a recent study we showed that a stable non-encapsulated derivative emerged during chronic osteomyelitis and displaced a more virulent CP8 parental strain which had been isolated several months before from the same infection site^9^ . The *S. aureus* derivative, which bore a frameshift mutation in *agrC* that caused loss of both RNAIII transcription and CP8 expression, was considered a microevolution endpoint adapted to bone. Lack of CP expression benefits the interaction between surface *S. aureus* adhesins and eukaryotic cell receptors thus promoting internalization of bacteria into the eukaryotic cell milieu^8^. Intracellular location, reduced capacity to cause inflammation^13,22^, increased biofilm production and SCV formation^23^ may explain why not only *S. aureus* pulmonary infection of cystic fibrosis patients^24^ but also *S. aureus* osteomyelitis become refractory to antibiotic treatment in spite of appropriate antibiotic treatment. From our bacterial collection we selected non-encapsulated *S. aureus* isolate HU-14 with a non-functional *agr* due to an IS*256* insertion in *agrC*. Although HU-14 may be considered a microevolution endpoint, in the present study we show that the passage of this isolate through blood in an experimental mouse model induced the reversion from a NT to a CP5 phenotype. Interestingly, it was not a true genetic reversion since IS*256* remained inserted at the same location in the *agrC* of the CP5 isogenic derivative. Therefore, loss of the *agr* functionality due to a stable mutation (in this case caused by an IS*256* insertion) may only be considered a microevolution endpoint at the precise niche where selection initially took place.

A variable number of relevant *S. aureus* isolates are non-reactive to antibodies anti-CP5 and anti-CP8 as phenotypically ascertained in vitro in the clinical bacteriology laboratory. Diverse mechanisms responsible for the loss CP5(8) expression have been reported, one of which is related to dysfunction of the main global regulator Agr^17^. There is ample evidence that *agr*-deficient strains may not only be selected during infection^25^ but also colonize healthy hosts^26^. In a study on 195 *S. aureus* isolates collected from different sources it was found that 49% of the isolates carried an inactive *agr* locus, as shown by the lack of a RNAIII transcript^27^. In the same study, interestingly, 55% of the strains with a non-functional *agr* produced capsule (CP5 or CP8). Such finding permits to speculate that the type of *S. aureus* derivative obtained after passage through the bloodstream described in the present report, which produce CP5 in the absence of a functional *agr*, may not be a rare event in clinical practice after all. CP5(8) is a strong candidate for construction of a multicomponent vaccine to prevent *S. aureus* severe infections^28^. Indeed, the most promising preparations contain CP conjugated to a toxoid plus other relevant antigens^16,29–31^. The finding that prevalent clone USA300 in the USA is non-encapsulated due to mutations in the capsular genes rather than in a capsule regulator^32^ appeared to cast doubt on the inclusion of CP as vaccine component. But on the other hand, many *S. aureus* clinical isolates carry a conserved *cap* cluster and, furthermore, it was recently demonstrated that clinical strains that do not produce CP in vitro are able to produce CP in vivo^20^. Our present study demonstrates the feasibility that *S. aureus* may recover in a stable manner CP5 production, which facilitates phagocytosis evasion once bacteria reach the bloodstream, in spite of a non-functional Agr. The results of our study suggest that the inclusion of CP in a multicomponent vaccine preparation should not be dismissed.

*Staphylococcus aureus* possesses an intricate regulatory system that governs the production of soluble virulence factors and exoenzymes. In addition to Agr, main regulators include a number of two-component systems, such as SaeRS, transcriptional factors^33^, such as SarA and its homologues^34^ and factor σB (*sigB*)^35^. Bioinformatic analysis of strains HU-14 and P3.1 sequences revealed that the sequences of the aforementioned regulators were conserved, with no differences between the parental strain and the derivative. Since it was recently demonstrated that CP production is up regulated by SigB we focused our attention on this regulator^36^. The SigB regulon is known to respond to different stress signals and accordingly regulates stress responses^33^. One of the marker genes upregulated by SigB is *asp23*^37^. An increase of SigB activity in P3.1 was suspected by noteworthy pigmentation of P3.1 colonies. Indeed, another known SigB-regulated trait is production of staphyloxanthin^38^. Further assessment revealed that P3.1 produced pigments whereas the parental strain HU-14 did not, as determined spectrophotometrically. In the present report, the significant increase in *asp23* expression revealed that the increase in SigB functionality was associated with the recovery of CP5 production. It is hypothesized that adaptation of *S. aureus* through a long-term process in the host may have led to reduced SigB expression and perhaps loss of the Agr function seen in HU-14. Experimental passage of this isolate through the bloodstream induced reacquisition of CP expression with parallel increase of SigB expression, as ascertained by increased *asp23* expression. In favor of this hypothesis, Marbach and coworkers^39^ has found that *S. aureus* infecting the bovine udder may not require *sigB* expression once it is established in the mammary gland of the cow with chronic mastitis. Interestingly, as we hypothesized before, loss of SigB expression in the infected cow was eventually fixed by mutation^8^. In this case the mutated gene was *rsbU*^39^.

Whole genome sequence analysis from reads obtained by Illumina sequencing did not reveal any genetic lesion directly or indirectly responsible for the observed SigB increased expression. Therefore, the presence of structural changes in P3.1 was then investigated by long-read sequencing. Hybrid (PacBio and Illumina) sequence analysis of the HU-14 and P3.1 chromosomes as well as of plasmid pCM05 suggested a structural change in P3.1 compared with HU-14, which consisted of an IS*256* excision located between *dnaG* (DNA primase) and *rpoD* (*sigA*). This preliminary observation was not surprising since mobile genetic elements are key drivers of evolution in *S. aureus*^40^. Giulieri and coworkers^41^ have indeed demonstrated that insertion of IS*256* elements enhances genetic diversity during infection thus representing an effective driver of within-host microevolution. Furthermore, insertion of IS*256* in *agrC* was found to promote increased fitness of *S. aureus* as a compensatory mechanism for the biological cost resulting from acquisition of a high number of multiresistant traits by hospital isolates^42^. In this regard, it is speculated that reacquisition of SigB activity and CP5 as well as staphyloxanthin production was perhaps achieved by restoration of SigA activity since IS*256* was excised without affecting the original conserved intergenic non-coding region between *dnaG* (DNA primase) and *rpoD* (*sigA*). In support of this hypothesis, it has been shown that the *sigB* operon is transcribed from at least two differentially controlled promoters, one of which is a putative σ^A^-dependent promoter termed *sigB*_P1_^43^. Therefore, *sigB* expression is driven by SigA, which leads to the transcription of the whole operon, as well as through autoregulation by SigB itself, leading to transcription of *rsbV* and *rsbW*^33^. Further research is required to better understand the complex mechanisms that permit concomitant reacquisition of CP5 and pigment production by *S. aureus* in the presence of an inactive *agr* and a functional *sigB* operon, which is known to be driven by SigA.

Altman and coworkers have shown that within-host loss of *agr* function was associated with increased genetic divergence between distinct *S. aureus* subclones^44^. In synthesis, whereas *agr* dysfunction is adaptive for survival in the infected bone, it may be counter adaptive outside such niche. As observed before, *agr* mutations do not tend persist in natural *S. aureus* populations^45^. In the present report we show that *S. aureus* can regain CP production when subjected to selection pressure in vivo through a mechanism that does not require RNAIII expression. Indeed, in a previous study it has been shown that genetic changes occurring in an *agr*-defective infecting *S. aureus* strain resulted in increased virulence in a murine model of bloodstream infection thus bypassing the *agr* mutation^44^. The *S. aureus* derivative obtained after passage through the bloodstream displayed a significant reduction in the capacity to trigger TNFα production and became less adapted to bone as ascertained by its decreased capacity to induce osteoclastogenesis. The *S. aureus* strain adapted to blood not only conserved but increased its capacity to produce biofilm. Recovery of at least two important traits such as CP5 and pigment production permit bacteria to evade innate immune mechanisms of defense in the blood^46–49^ and readapt to a new environment elsewhere in the host with the potential to cause methastatic infection. The fact that *S. aureus* can adapt to blood by regaining the capacity to produce CP5 and pigments does not necessarily mean that virulence should increase. P3.1 exhibited a trend to be more virulent, as ascertained in the mouse model of intraperitoneal challenge, but the differences were not significant. This was not a surprising fact since the Agr remained non-functional and many other Agr-regulated virulence factors are not being expressed, such as the hemolysins. Therefore, as much as *S. aureus* is a multifactorial pathogen, it is speculated that its adaptation to a given niche in the host is most probably multifactorial too. In the present study, modification of virulence factor expression due to increased SigB activity was enough to reduce adaptation to bone but not to increase virulence in a significant fashion.

## Conclusion

Here we demonstrated that *S. aureus* adapted to infected bone can regain the ability to produce CP, even in the presence of a mutated, non-functional Agr when transferred to the bloodstream. *S. aureus* organisms that escape the infected bone can potentially become a threat because they can recover in a stable manner the expression of traits that permit survival in blood, such as CP and staphyloxanthin. Therefore, a supposedly harmless endpoint-of-microevolution may turn back into a dangerous infective derivative. Given that CP is one of the factors identified in this microevolution process, our findings support the idea that *S. aureus* CP should not be dismissed as a multifactorial vaccine candidate component.

## Materials and methods

### Bacterial strains and cultures

*Staphylococcus aureus* clinical isolate HU-14 from our strain collection was originally recovered in 2005 from a 34-year old male patient with chronic osteomyelitis of the right femur with a prosthetic implant (“Hospital Ramón Carrillo”, Buenos Aires, Argentina). Species was confirmed by a species-specific PCR^50^. Strain HU-14 is ST5, CC5, SCC*mec* type 1, Agr type II and Spa t149^51^. All strains utilized in the study were kept frozen in trypticase soy broth (TSB) with 20% glycerol at − 80 °C and *S. aureus* was routinely cultured at 37 °C for 24 h on TSB unless otherwise indicated. In addition, *S. aureus* strain 6850 was used as a qRT-PCR positive control for Agr and SigB functionality testing. *S. aureus* strains RN6911 and SH1000 were utilized as negative and positive controls, respectively, for staphyloxanthin production. *S. aureus* strains Sa113 and MBD034 were utilized as positive and negative controls, respectively, for PIA production. Strain HU-8 (CC5, SCC*mec* type 1, Agr type II, Spa t149 and produces CP5), which bears a functional *agrC,* was also utilized as control.

### Mouse models

CF1 outbred mice were bred and maintained in the vivarium of the “Instituto de Investigaciones en Microbiología y Parasitología Médica” (IMPaM-Universidad de Buenos Aires-CONICET, Buenos Aires, Argentina). Animal care was in accordance with the recommendations of the guidelines set forth by the 11th report of the BVAAWF/FRAME/RSPCA/UFAW Joint Working Group on Refinement^52^. The animal research protocols utilized in this study were approved by the “Comité Institucional para el Uso y Cuidado de los Animales de Laboratorio,” through resolutions No. 885/19 issued on June 5, 2019 and N^o^ 2780/18 issued on February 5, 2019 by the “Consejo Directivo de la Facultad de Medicina, Universidad de Buenos Aires,” Argentina. The experimental scheme of the mouse bacteriemia model is shown in Fig. 1. Passage of *S. aureus* strain HU-14 through blood was performed on groups of 4 male mice in each cycle. An inoculum of 1 × 10^8^ CFU in 100 µl of physiologic saline solution (PSS) was injected in mice by the ip route. After 24 h mice were sacrificed by exposure to CO_2_ and blood was drawn by cardiac puncture and quantitatively plated on trypticase soy agar (TSA). Plates were incubated for 24 h at 37 °C and colonies were suspended PSS to ca. 1 × 10^9^ CFU to prepare a 10^8^ CFU inoculum to be re-injected in the next group of mice. This cycle was repeated seven times. To assess the differential virulence of the strains under investigation mortality curves were compared. Groups of 10 CF1 outbred mice were injected with 1 × 10^8^ CFU of the strain to be tested suspended in 0.5 ml of 2% (w/v) Brewer´s yeast (Sigma Chemical Co.) in TSB broth. Mortality and body weight were assessed daily for 5 days. Surviving mice were euthanized by cervical dislocation. The Kaplan–Meier mortality curve was obtained using the Graph-Pad Prism software (GraphPad Software, Inc., La Jolla, USA; version 6.00).

### Illumina whole genome sequencing and analysis

DNA was extracted directly from plated microbial cultures using the Nextera DNA Flex Microbial Colony Extraction protocol (Illumina, San Diego, CA, USA). Library preparation was performed according to the Nextera DNA Flex Library Prep Kit using Nextera DNA 24 CD Indexes (Illumina, San Diego, CA, USA). Paired-end whole genome sequencing was performed in a MiSeq instrument (Illumina, San Diego, CA, USA) using the MiSeq Reagent kit v2 150 bp. The resulting fastq files were analyzed with the FastQC High Throughput Sequence QC Report Version 0.11.7 (https://www.bioinformatics.babraham.ac.uk/projects/). Only isolates that fulfilled the quality parameters of the FastQC software (e.g. per base sequence quality, per tile sequence quality, per sequence GC content) were used for analysis. The FastQ files were assembled using SPAdes v3.10.1^53^. The genome was annotated using Prokka v1.14.5^54^ and variant calling was performed using Snippy v3.2 (https://github.com/tseemann/snippy). The sequences analyzed in this study have been deposited in the European Nucleotide Archive (ENA) under Bioproject PRJEB35732.

### Pacific biosciences genome sequencing and analysis

High molecular weight (HMW) gDNA was extracted from HU-14 and P3.1. Bacteria were grown overnight on TSA plates, then gDNA was extracted using Qiagen’s MagAttract HMW DNA Kit (Qiagen, Manchester, UK) according to the manufacturer’s instructions for Gram Positive species, using 20 µl of lysostaphin (10 mg/ml) instead of lysozyme. The extracted HMW gDNA was sent to the Centre for Genomic Research at the University of Liverpool, UK, where long-read sequencing and base modification analysis were conducted on the Pacific Biosciences (Pacific Biosciences, Menlo Park, CA, USA) Sequel system. The raw subreads for each sample were filtered to 200 × coverage of the *S. aureus* genome using Filtlong v0.2.0 (available at https://github.com/rrwick/Filtlong) and then corrected using Canu v1.9^55^. Hybrid de novo assemblies were produced for both samples by Unicycler v0.4.8^56^, using the PacBio long reads and Illumina short reads. In hybrid mode, Unicycler uses SPAdes v3.14.0^53^ to assemble the short reads, then bridges the SPAdes contigs and closes the genome using the long reads with minimap and miniasm (built-in Unicycler v0.4.8 versions)^57^, followed by long-read polishing with Racon v1.4.11^58^ and short-read polishing with Pilon v1.22^59^. The completeness of the closed genome sequences was assessed using BUSCO v4.0.2^60^. The “bacteria” database was used to search the genomes for 120 universal bacterial genes, and the more specific “Bacillales” database was used to search for 450 genes common to all Bacillales species. Prokka v1.14.5^54^ was used to annotate the closed genome sequences, using the protein sequences from the *S. aureus* strain N315 (NC_00275.2) as a reference. The annotated GenBank format files were then used to call SNPs between the two samples with Snippy v4.5.1 (https://github.com/tseemann/snippy); the HU-14 GenBank file was used as the reference with the P3.1 Illumina reads, and vice-versa. The plasmid sequence for each sample was compared to the PLSDB plasmid database v.2019_10_07^61^ (11), and annotated with Prokka (1.14.5)^54^ to confirm its identity. The closed chromosome and plasmid sequences from each sample were rotated to start with the same genes (*dnaA* for the chromosome, *repA* for the plasmid) and aligned using progressiveMauve (build version: Sep 16 2015). The output files were inspected visually for any rearrangements using the progressiveMauve GUI.

### Phenotypic studies

CP5 production was evaluated by colony immunoblot on TSA plates as described previously^62^. Phenotypic expression of α- and β-hemolysin was performed by evaluating the production of the hemolysis in rabbit and goat blood agar (α- and β-hemolysin, respectively). Proteolytic activity was assessed in TSA supplemented with 10% whole milk. After 18 h incubation at 37 °C the hemolytic and proteolytic halos were evaluated, respectively. Ethanolic extracts of the carotenoid pigment from bacterial suspensions with equal optical density (OD) at 600 nm were quantified spectrophotometrically at 450 nm^63^. The quantitative assessment of biofilm formation was performed as previously described with modifications^64^. Briefly, *S. aureus* strains were grown for 18 h and diluted 1:100 in TSB supplemented with 0.25% of glucose (TSBg). After 24 h of static incubation at 37 °C in 96-well microtiter plates, bacterial growth in each well was measured by optical density reading at 595 nm (OD_G_) using a microplate reader (Multiskan EX). Then, the wells were washed two times with PBS, the biofilms were fixed with 100% methanol for 15 min, stained with 0.5% crystal violet for 20 min, and washed twice again gently under running tap water. The amount of biofilm biomass was measured after addition of 30% glacial acetic acid by OD reading at 595 nm (OD_B_). The biofilm biomass was expressed relative to the final cell density measured prior to crystal violet staining (biofilm OD_B_/OD_G_). Three independent experiments were performed in sixtuplicate measurements. PIA production was assessed by ELISA according to a protocol described elsewhere^64^. Briefly, the procedure was similar to that described above for biofilm except that, after blocking solution removal,100 μl per well was added of a 75 ng/ml wheat germ agglutinin (WGA)-HRP conjugate, a lectin that binds to PIA sugar residues.

### Real-time quantitative reverse transcription PCR (qRT-PCR)

Bacterial RNA was extracted from *S. aureus* grown in TSB until post-exponential phase, using TRIzol Reagent (Invitrogen Life Technologies), according to the manufacturer’s protocol. RNA was subjected to DNAse treatment using a RQ1 RNAse free DNAse (Promega). cDNA synthesis was performed with an ImProm-II Reverse Transcriptase kit (Promega). qRT-PCR for RNAIII, *agrA* and *asp23* expression were performed using the primers and conditions described in a previous publication^9^ and the SYBR Green PCR Master Mix (Applied Biosystems) equipment and kits. The *16S* gene was used to normalize data. The (− ΔCt) value represents the difference in threshold cycle (Ct) between the target and control(*16S*) genes^65^.

### Osteoclastogenesis assay and TNFα detection

To determine the differential capacity of strains HU-14 and P3.1 to trigger osteoclastogenesis RAW 264.7 cells were plated on coverslips (2,5 × 104 cells/well, 24 well plate) in the presence of M-CSF 30 ng/ml (MACS Miltenyi Biotec) and 107 CFU/ml or each *S. aureus* strain or media alone. At 48 h after stimulation cells were fixed in 4% paraformaldehyde and stained for Tartrate-Resistant Acid Phosphatase (TRAP) (Sigma-Aldrich, St. Louis, MO, USA). Multinucleated (more than three nuclei), TRAP-positive cells were defined as osteoclasts and counted^66^. TNFα concentrations were determined quantitatively in culture supernatants collected at 24 h after stimulation by ELISA using specific antibody pairs (Beckton-Dickinson) as described previously^67^.

### Statistical considerations

Multiple data statistical comparison was performed by ordinary one-way ANOVA using the Tukey´s multi comparisons test and the Bartlett-Forsythe post-test. Statistical comparison of two data groups with normal distribution was performed with the Student *t* test for unpaired samples. Statistical comparison of two data groups with non-normal distribution was performed with the Mann–Whitney test. P < 0.05 was considered statistically significant. Graph-Pad Prism software (GraphPad Software, Inc., La Jolla, USA; version 6.00) was used for all statistical analyses.

## Supplementary information

Supplementary file 1.

Supplementary file 2.

Supplementary file 3.

## Data Availability

The sequences analyzed in this report have been deposited in the European Nucleotide Archive (ENA) under Bioproject PRJEB35732. Other datasets generated and/or analyzed during the current study are available from the corresponding author upon request.
