# Supplementary figures and images for: Acapsular Staphylococcus aureus with a non-functional agr regains capsule expression after passage through the bloodstream in a bacteremia mouse model
Source: Sci Rep. 2020 Aug 24;10:14108. doi: 10.1038/s41598-020-70671-1 (PMC7445255; doi:10.1038/s41598-020-70671-1)

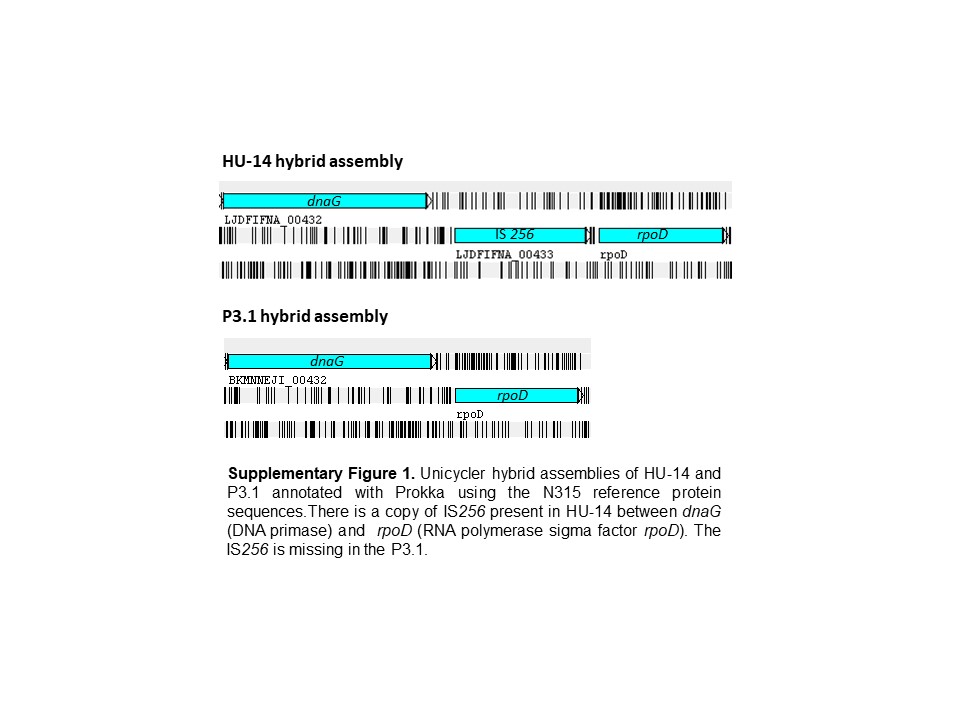

Supplement: Supplementary file 1 [file 41598_2020_70671_MOESM1_ESM.jpg]

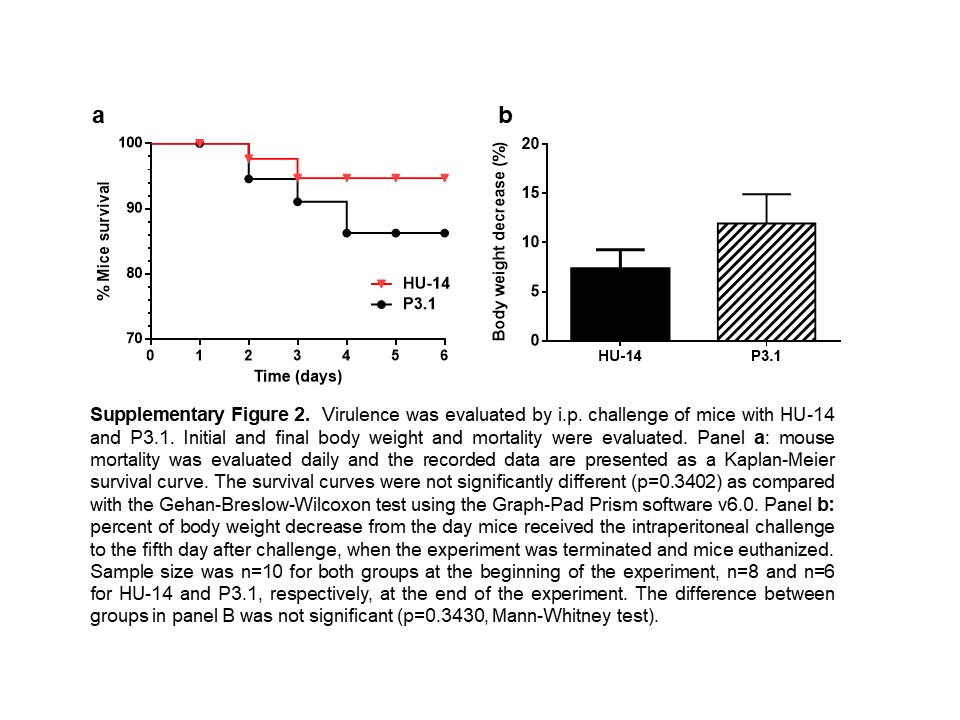

Supplement: Supplementary file 2 [file 41598_2020_70671_MOESM2_ESM.jpg]

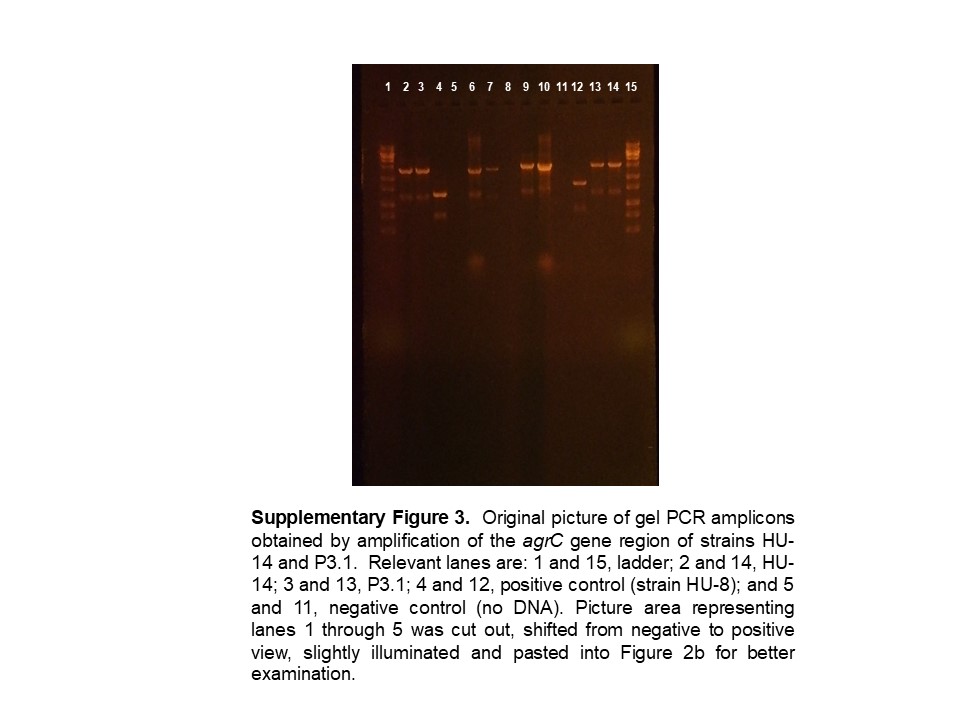

Supplement: Supplementary file 3 [file 41598_2020_70671_MOESM3_ESM.jpg]
